# Understanding the biology and use of TNF therapy in jia-clinical outcomes

**DOI:** 10.1186/1546-0096-12-S1-P137

**Published:** 2014-09-17

**Authors:** Daniel Lovell, Anne Johnson, Yuki Kimura, Steve Spalding, Paula Morris, Beth Gottlieb, Karen Onel, Judyann Olson, Barbara Edelheit, Michael Shisov, Lawrence Jung, Elaine Cassidy, Sampath Prahalad, Murray Passo, Tim Beukelman, Jay Mehta, Kara Schmidt, Dirk Foell, Claas Hinze, Bin Huang, Edward Giannini

**Affiliations:** 1 Cincinnati Children's Hospital Medical Center, Cincinnati, Ohio, USA; 2 Joseph M Sanzari Children’s Hospital, Hackensack, New Jersey, USA; 3 Cleveland Clinic Foundation, Cleveland, Ohio, USA; 4 Arkansas Children's Hospital, Little Rock, Arkansas, USA; 5 Cohen Children's Medical Center, New Hyde Park, New York, USA; 6 Comer Children's Hospital, Chicago, Illinois, USA; 7 Children's Hospital of Wisconson, Milwaukee, Wisconsin, USA; 8 Connecticut Children's Medical Center, Hartford, Connecticut, USA; 9 Phoenix Children's Hospital, Phoenix, Arizona, USA; 10 Children's National Medical Center, Washington, D.C., USA; 11 Children's Hospital of Pittsburgh, Pittsburgh, Pennsylvania, USA; 12 Emory University, Atlanta, Georgia, USA; 13 Medical University of South Carolina, Charlestown, South Carolina, USA; 14 University of Alabama at Birmingham, Birmingham, Alabama, USA; 15 Children's Hospital at Montifiore, Bronx, New York, USA; 16 Kosair Children's Hospital, Louisville, Kentucky, USA; 17 University of Muenster, Muenster, Germany

## Introduction

Treatment with anti-TNF therapies (anti-TNF) for polyarticular forms (extended oligo, Poly RF +/-) of JIA (PF-JIA) results in >50% demonstrating clinical inactive disease (CID).

## Objectives

The aims of this study were to perform the first prospective, mulicenter trial to determine the frequency, timing and predictors of flare upon withdrawal of anti-TNF in PF-JIA in CID.

## Methods

In 16 centers 137 children with PF-JIA in CID on anti-TNF were enrolled and prospectively followed. If CID was maintained for the first 6 study mos, then anti-TNF was stopped and the patients were followed prospectively by protocol. Background meds were stable.

## Results

The study population included 18 (13%) extended oligoarticular, 17 (12%) RF+ Poly and 102 (75%) RF- Poly JIA patients. At enrollment, age (mean/median/range) was 11.3/11.6/3.4-20.1 yrs; disease duration was 5.0/4.1/0.6-18.6 yrs; 103 (75%) were females and 64 (47%) were ANA+. Duration of CID at baseline was 1.2/0.5/1 day-12.1 yrs. Anti-TNF was etanercept in 106 (77%), 25 (18%) adalimumab and 6 (5%) infliximab. 40% were on MTX at baseline (mean/median dose 0.4/0.4 mg/kg/wk).

17% were unable to maintain CID for the first 6 months despite stable background medications. For the extended oligo, Poly RF – and Poly RF+ categories 94%, 82% and 60%, respectively, maintained CID for the first 6 months (chi-square 6.7, p 0.03). ANA status, MTX use, and type of anti-TNF were not associated with the ability to maintain CID (chi-square p values 0.48, 0.14, and 0.75, respectively).

Upon stopping the anti-TNF therapy, the mean time to flare was 18.3 months with a median of 26 months (range 9-32 months). Longer disease duration at baseline was associated with an increasing risk of flare with stopping anti-TNF therapy (chi square 5.62, p = 0.017). Background MTX significantly decreased the risk of flare (p= 0.05) and significantly increased the time to flare (p = 0.05). JIA subtype was significantly associated with both risk of flaring (p=0.02) and time to flare (p= 0.04) with RF+ Poly flaring less frequently than either RF- or Extended oligo which seem similar. RF+ patients were significantly less likely to flare than RF- (p= 0.02). Age, gender, ANA status, duration of CID did not predict risk of or time to flare.

## Conclusion

In these patients with Polyarticular forms of JIA in CID for ≥6 mos, upon stopping the anti-TNF therapy, 70% will experience a flare within 3.25 years but ≥ 50% will maintain CID for ≥17 months. Continuing background MTX both decreases the risk of flare and increases the time to flare. Disease duration and JIA subtype are the only predictive clinical parameters. Duration of CID was NOT predictive of risk of flare after stopping anti-TNF therapy.

## Disclosure of interest

D. Lovell: consultant for: Roche, Genetech, Jannsen, AstraZeneca, Pfizer, Novartis, Abbott, Forest Research, BMS. Speaker Bureau of: Novartis, Roche, Genetech. A. Johnson: none declared. Y. Kimura: Consultant for: Novartis.

S. Spalding: none declared. P. Morris: none declared. B. Gottlieb: none declared. K. Onel: none declared. J. Olson: consultant for: Abbott. B. Edelheit: none declared. M. Shisov: none declared. L. Jung: none declared. E. Cassidy: none declared. S. Prahalad: none declared. M. Passo: none declared. T. Beukelman: grant / research support from Pfizer, consultant for Novartis, Genentech, UCB. J. Mehta: none declared. K. Schmidt: none declared.

D. Foell: none declared. C. Hinze: none declared. B. Huang: none declared. E. Giannini: none declared.

